# Conserved mechanism of Xrn1 regulation by glycolytic flux and protein aggregation

**DOI:** 10.1016/j.heliyon.2024.e38786

**Published:** 2024-10-01

**Authors:** Satyendra Mondal, Jakub Zahumensky, Petra Vesela, Jan Malinsky

**Affiliations:** Department of Functional Organization of Biomembranes, Institute of Experimental Medicine, Academy of Sciences of the Czech Republic, 142 20, Prague, Czech Republic

**Keywords:** Eisosome, Xrn1, Exoribonuclease, Yeast, Glycolysis, SH3-like domain

## Abstract

The regulation of gene expression in eukaryotes relies largely on the action of exoribonucleases, evolutionarily conserved enzymes that digest decapped messenger RNAs in the 5’-3’ direction. The activity of Xrn1, the major yeast exoribonuclease, is regulated by targeted changes in its cellular localisation in direct response to the cell’s metabolic state. When fermentable carbon sources are available, active Xrn1 is diffusely localised in the cytosol. Upon depletion of these sources, Xrn1 is sequestered at the plasma membrane-associated protein complex, the eisosome, and becomes inactive. Although this phenomenon has been described previously, the molecular mechanisms underlying these changes remain unknown. We report that the binding of Xrn1 to the plasma membrane is subject to glycolytic flux, rather than the availability of a fermentable carbon source, is independent of TORC1 activity and requires the core eisosomal proteins Pil1 and Lsp1. We identify the SH3-like domain of the Xrn1 protein as a putative interaction domain. In addition, we show that when expressed in *Saccharomyces cerevisiae*, the human orthologue of Xrn1 mirrors its yeast counterpart, i.e., it segregates to the eisosome under conditions of halted glycolysis. Our results not only advance our understanding of Xrn1 regulation but also indicate that this regulatory principle is conserved from yeast to humans.

## Highlights

• Eisosome core components are required for the plasma membrane association of Xrn1.
• Binding of Xrn1 to the membrane is mediated by the C-terminal region of the protein.
• Glycolytic flux, not a fermentable carbon source, is crucial for Xrn1 activation.
• Glycolysis-dependent Xrn1 sequestration is likely conserved in higher eukaryotes.

## 1 Introduction

The regulation of gene expression via mRNA degradation is a widely conserved process across the eukaryotic domain. The primary mechanism for mRNA turnover involves exoribonuclease-mediated 5’-3’ digestion following the removal of the protective 5’-end cap [[1], [2], [3], [4]]. This is complemented by 3’-5’ mRNA decay activity provided by the exosome-SKI complex [5,6]. In each case, the mRNA is first trimmed at the poly-A 3' end, a process called deadenylation [7]. Although alternative ways of mRNA degradation have been described [4,8], the absence of both Xrn1 and exosome activity in yeast is lethal [9,10].

The localisation and activity of the dominant yeast 5’-3’ exoribonuclease Xrn1 are closely linked to the metabolic state of the cell [11,12]. During exponential growth, when fermentable sugars are abundant, Xrn1 is diffusely localised throughout the cytosol and executes large-scale mRNA turnover. When glycolysis stalls due to limited substrate availability, a significant fraction of the enzyme accumulates at specialised membraneless ribonucleoprotein particles called processing bodies (P-bodies) and its overall activity decreases [13]. Following the diauxic shift to nonfermentable carbon sources and respiration, Xrn1 segregates to the eisosome, a plasma membrane-associated protein scaffold, where it becomes completely inactivated [11,12]. Upon the reintroduction of glucose, Xrn1 is released from the eisosome and redistributed into the cytosol [11], where it reactivates within minutes [12]. Although the *XRN1* gene is not essential in yeast, its absence leads to serious cellular challenges due to the accumulation of partially deadenylated, decapped, nondegraded mRNAs [14], resulting in reduced population growth and cell cycle progression [15].

Eisosomes are large plasma membrane-associated protein complexes conserved throughout evolution, particularly in the *Ascomycota* phylum ([16,17] and references therein), which play important roles in adaptation to various kinds of environmental stress [[18], [19], [20], [21]]. Structurally, eisosomes are composed of BAR (Bin/Amphiphysin/Rvs) domain-containing Pil1 and Lsp1 proteins, which spontaneously assemble into hemitubular structures that are more or less evenly distributed over the cell surface [16,[22], [23], [24], [25]]. To date, two dozen proteins have been shown to associate with eisosomes, either through direct interaction with the hemitubules or through accumulation at the plasma membrane microdomain bound by their protein structures [19,21,26,27]. Although the functional implications of Xrn1 binding to eisosomes have been documented [12], the underlying forces and mechanisms driving this process have not yet been elucidated.

In this study, we demonstrate that the binding of Xrn1 to the plasma membrane requires the core eisosomal proteins Pil1 and Lsp1 and identify a putative membrane-binding domain within the Xrn1 protein sequence. We also show that halting the glycolytic flux is both necessary and sufficient to induce the association of Xrn1 with the eisosome and that this mechanism of plasma membrane binding is followed by the human *Hs*Xrn1 protein when it is expressed in *Saccharomyces cerevisiae* lacking its native orthologue. This indicates that the membrane-associated regulation of Xrn1 activity may be conserved from yeast to humans.

## 2 Materials and methods

### 2.1 Strains and growth conditions

All *S. cerevisiae* strains used in this study are listed in Table 1. If not stated otherwise, yeast cells were grown in a synthetic complete medium (SC; 0.17 % yeast nitrogen base without amino acids and ammonium sulphate, 0.5 % ammonium sulphate, 2 % D-glucose, amino acids) at 28 °C and 220 rpm in an incubated shaker. The yeast strains expressing the non-integrative centromeric plasmids pUG35 and YCplac111 (see below) were grown in synthetic dropout Ura- and Leu-media, respectively. Overnight cell cultures were inoculated into fresh media and adjusted to the optical density of OD_600_ = 0.2. The culture was grown for 12 (to late-exponential phase) or 30 h (post-diauxic) according to the type of experiment. For the propagation of plasmids, the *Escherichia coli* strain XL1-Blue (Stratagene, San Diego, CA, USA) was used. Bacterial strains were grown in LB medium (1 % tryptone, 0.5 % yeast extract, 1 % NaCl) supplemented with ampicillin (100 μg/ml) for transformant selection.

**Table 1:** **Yeast strains used in the study.** Introduced modifications are highlighted in bold.

Strain	Genotype	Source
BY4741, wild-type	*MATa; his3*Δ*1; leu2*Δ*0; met17*Δ*0; ura3*Δ*0*	Euroscarf
BY4742, wild-type	*MATα; his3*Δ*1; leu2*Δ*0; lys2*Δ*0; ura3*Δ*0*	Euroscarf
*lsp1*Δ	BY4742; ***lsp1*Δ***::kanMX4*	Euroscarf
*nce102*Δ	BY4741; ***nce102*Δ***::kanMX4*	Euroscarf
*seg1*Δa	BY4741; ***seg1*Δ***::kanMX4*	Euroscarf
*seg1*Δα	BY4742; ***seg1*Δ***::kanMX4*	Euroscarf
*ski2*Δ	BY4741; ***ski2*Δ***::kanMX4*	Euroscarf
*xrn1*Δ	BY4741; ***xrn1*Δ***::kanMX4*	Euroscarf
*pil1*Δ	BY4741; ***pil1*Δ**::*pFA6a::natMX6*	This study
*pil1*Δ*lsp1*Δ	Y431 x Y432; isogenic to BY4741 except ***pil1*Δ***::kanMX4;****lsp1*Δ***::kanMX4*	This study
*pil1*Δ*nce102*Δ	Y087 x Y088; isogenic to BY4741 except ***pil1*Δ***::kanMX4;****nce102*Δ***::kanMX4*	This study
*seg1*Δ*pil1*Δ	Y373 x Y374; isogenic to BY4742 except ***seg1*Δ***::kanMX4;****pil1*Δ***::kanMX4*	This study
*seg1*Δ*nce102*Δ*pil1*Δ	Y429 x Y435; isogenic to BY4741except ***seg1*Δ***::kanMX4;****nce102*Δ***::kanMX4;****pil1*Δ***::kanMX4*	This study
Y087	*pil1*Δ; ***NCE102-mRFP****::URA3* (YIp211)	This study
Y088	*nce102*Δ; ***PIL1-GFP****::LEU2* (YIp128)	This study
Y373	*pil1*Δ; ***SEG1-GFP****::LEU2* (YIp128)	This study
Y374	*seg1*Δα; ***Pil1-mRFP****::URA3* (YIp211)	This study
Y429	*seg1*Δ*pil1*Δ; ***NCE102-mRFP****::URA3* (YIp211)	This study
Y431	*lsp1*Δ; ***PIL1-GFP****::URA3* (YIp211)	This study
Y432	*pil1*Δ; ***LSP1-mRFP****::LEU2* (YIp128)	This study
Y435	*pil1*Δ*nce102*Δ; ***SEG1-GFP****::LEU2* (YIp128)	This study
Y1286	Y1288; ***pil1*Δ**::*pFA6a::natMX6*	This study
Y1288	BY4741; ***XRN1-GFP****::SpHIS5* (pKT128)	This study
Y1319	*pil1*Δ*lsp1*Δ; ***XRN1-GFP****::SpHIS5* (pKT128)	This study
Y1432	*ski2*Δ; ***XRN1***_***1-1241***_***-GFP****::SpHIS5* (pKT128)	This study
Y1433	*ski2*Δ; ***XRN1***_***1-1132***_***-GFP****::SpHIS5* (pKT128)	This study
Y1439	BY4741; ***XRN1***_***1-1241***_***-GFP****::SpHIS5* (pKT128)	This study
Y1440	BY4741; ***XRN1***_***1-1132***_***-GFP****::SpHIS5* (pKT128)	This study
Y1449	Y1286; *mRFP::LEU2* (YCp111)	This study
Y1451	Y1286; ***PIL1-mRFP****::LEU2* (YCp111)	This study
Y1452	*nce102*Δ; ***XRN1-GFP****::SpHIS5* (pKT128)	This study
Y1453	*seg1*Δ*nce102*Δ*pil1*Δ; ***XRN1-GFP****::SpHIS5* (pKT128)	This study
Y1470	*xrn1*Δ; ***HsXRN1-GFP****::URA3* (pUG35)	This study
Y1487	BY4741; ***XRN1***_***1133-1528***_***-GFP****::URA3* (pUG35)	This study
Y1488	*xrn1*Δ; ***XRN1***_***1133-1528***_***-GFP****::URA3* (pUG35)	This study
Y1490	*seg1*Δa; ***XRN1-GFP****::SpHIS5* (pKT128)	This study
Y1506	Y1286; ***LSP1-mRFP****::LEU2* (YIp128)	This study
Y1544	*xrn1*Δ; ***XRN1-GFP****::URA3* (pUG35)	This study
Y1565	Y1319; ***PIL1-mRFP****::LEU2* (YCp111)	This study
Y1578	Y1319; ***PIL1(5P)-mRFP****::LEU2* (YCp111)	This study
Y1581	*ski2*Δ; ***XRN1-GFP****::SpHIS5* (pKT128)	This study
Y1582	Y1288; ***lsp1*Δ**::*pFA6a::natMX6*	This study

A growth assay was conducted to test the viability in *ski2*Δ mutant expressing full-length Xrn1 (Y1581) and two C-terminally-truncated Xrn1 versions (Y1432 and Y1433, respectively). Three biological replicates were grown starting from OD_600_ = 0.2 in 50 ml SC media. The OD_600_ was measured every hour in the first 15 h of growth, following measurements in 3-h intervals. The resulting growth curves were plotted using the Systat SigmaPlot 15.0 software.

### 2.2 Mating and sporulation

The *pil1*Δ*lsp1*Δ and *seg1*Δ*nce102*Δ*pil1*Δ strains were prepared by mating (see Table 1 for details), in which the parental strains carried a complementary combination of deleted and fluorescently tagged genes. This was followed by sporulation on solid Fowell media (0.1 M potassium acetate, 0.1 % yeast extract, 0.05 % D-glucose, 2 % agar) and tetrad dissection using Singer micromanipulator (Singer Instruments, UK). The deletion mutants were selected with the help of fluorescence microscopy as non-fluorescent colonies. The absence of respective genes was then verified by PCR using gene-specific primers.

### 2.3 Plasmids/PCR cassettes preparation and transformations

All the vectors used in this study were delivered to the yeast cells by LiAc (lithium acetate)/SS (single stranded) carrier DNA/PEG (polyethylene glycol) transformation method as described previously [28]. The appropriate transformants were selected with either an antibiotic resistance marker or an auxotrophic marker on plates with respective media (Table 1) and verified via colony PCR and confocal microscopy. The yeast strain codes in the brackets in the following text denote the resulting strains.

To enable the expression under the native promoter, chromosomal tagging of the full-length or C-terminally truncated variants of *XRN1* with *GFP* was achieved as follows: the respective transformation cassette was amplified by PCR from the pKT128 plasmid carrying besides the gene for GFP also the *HIS5* gene from *Schizosaccharomyces pombe* as a selection marker (complementary to *HIS3* gene of *S. cerevisiae*) [29]. Forward primers were designed to contain a part complementary with either the end (excluding the stop codon; for full-length version) or the sequence immediately preceding the truncation point inside the *XRN1* gene (uppercase) and upstream of the *GFP* sequence (lowercase). Reverse primers contained part of either the 3’-UTR of the *XRN1* gene (for full-length version) or the sequence immediately following the truncation point inside the *XRN1* gene (uppercase) and a part complementary to the *HIS5* selection marker gene (lowercase). Primers for the labelling of the full-length *XRN1*: forward – CCAAAGTCACAAAGCAATGCTGCTGACCGTGATAATAAAAAAGACGAATCTACTcggatcggtgacggtgctgg; reverse – GGTCTCAGATATACTATTAAAGTAACCTCGAATATACTTCGTTTTTAGTCGTATGcatcgatgaattcgagctcg. The cassette was transformed into the yeast wild-type strain (Y1288; Table 1), *lsp1*Δ (Y1582), *pil1*Δ (Y1286), *pil1*Δ*lsp1*Δ (Y1319), *ski2*Δ (Y1581), *seg1*Δ (Y1490), *nce102*Δ (Y1452), and *seg1*Δ*nce102*Δ*pil1*Δ (Y1453). Primers for the labelling of the truncated Xrn1_1-1241_: forward – TGCCTCTTTCTTATTGAATATTACTAACAGGCAGTTCATTTATcggatcggtgacggtgctgg; reverse – CTATTAAAGTAACCTCGAATATACTTCGTTTTTAGTCGTATGTTcatcgatgaattcgagctcg. Primers for the labelling of the truncated Xrn1_1-1132_: forward – CTAAGACATCGATTGCTGCCGTGGAAGATCATATCATGAAATACGCAGCTcggatcggtgacggtgctgg; reverse – CAGCCTCACGAGGAACTTTGGCTAACTGTTTTCTTTCATGACCTTCGATGcatcgatgaattcgagctcg. These two cassettes for the truncated proteins were transformed into the wild-type (to obtain Y1439, and Y1440, respectively) and *ski2*Δ strains (resulting in Y1432 and Y1433, respectively).

The **YIplac128-*LSP1-mRFP*-*LEU2*** plasmid was constructed as follows: The *LSP1* gene was amplified by PCR from the *S. cerevisiae* genomic DNA using the forward primer AAACTCGAGACACAAGAATGCACAG and reverse primer AAAGGATCCCATGTTTTCAGAACCG. The obtained fragment was ligated into YIplac128-*mRFP-LEU2* plasmid using the XhoI/BamHI restriction sites. The plasmid was linearised by digestion with the PmlI restriction enzyme and transformed into the *XRN1-GFP*-expressing *pil1*Δ strain (Y1506).

For exogenous expression of wild-type Pil1 and the Pil1 carrying an exchange of five prolines in the N-terminal region P21-P25 for a flexible linker sequence GSGSG (designated henceforth as Pil1(5P)), centromeric non-integrative expression vectors were constructed using the YCplac111-*mRFP-LEU2* plasmid. **YCplac111-*PIL1*(prom)-*PIL1-mRFP-LEU2*:** the *PIL1* gene was amplified from the YIplac128-*PIL1*-mRFP plasmid (lab collection) using the forward primer CGTAGTCGACATGCACAGAACTTACTC and reverse primer CATAGGATCCAGCTGTTGTTTGTTGGG, and the amplification product was inserted into the YCplac111-*PIL1*(prom)-*SpPIL1-mRFP* expression vector (described in Ref. [30]) in exchange for the *SpPIL1* using the unique restriction sites of SalI and BamHI. The mRFP-labelled wild-type *PIL1* was co-expressed with the genomic *XRN1-GFP* by transforming the plasmid into the *pil1*Δ (Y1451) and *pil1*Δ*lsp1*Δ strain (Y1565). **YCplac111-*PIL1*(prom)-*PIL1(5P)-mRFP-LEU2***: a mutated *PIL1* gene segment sequence with codons for the five N-terminal prolines CCA CCG CCA CCA CCA (DNA base pairs 61.75) exchanged for GGT TCT GGT TCA GGT, which codes for a flexible linker (GSGSG), was purchased from Eurofins Genomics. It was inserted into the expression vector YCplac111-*PIL1*(prom)-*PIL1-mRFP-LEU2* using the unique restriction sites of SalI and XbaI in exchange for the wild-type allele. The Pil1(5P) was co-expressed with genomic Xrn1-GFP by transforming the plasmid into the *pil1*Δ*lsp1*Δ (Y1578) strain. The empty **YCplac111-*mRFP-LEU2*** plasmid (which does not contain a promoter sequence) was transformed into Xrn1-GFP-expressing *pil1*Δ (Y1449) strain as a negative control.

For exogenous expression of yeast and human *XRN1* (*HsXRN1*), the centromeric non-integrative plasmid vector pUG35-*MET17*(prom)-*GFP-URA3* was used. **pUG35-*MET17*(prom)-*XRN1-GFP-URA3*:** the native *XRN1* gene was amplified from the genomic DNA using the forward primer CTCTAGAACTAGTGGATCCATGGGTATTCCAAAATTTTTCAGGTAC and the reverse primer GGTATCGATAAGCTTGATATCAGTAGATTCGTCTTTTTTATTATCACGG and inserted into the pUG35 plasmid after the *MET17* promoter sequence using the unique restriction sites of BamHI and HindIII at the multi-cloning site downstream of the *MET17* promoter and upstream of the *GFP-*coding sequence. **pUG35-*MET17*(prom)-*HsXRN1-GFP-URA3*:** for the *HsXRN1* expression, the coding sequence of *HsXRN1* was amplified from a cDNA library from the DMSO-treated control BEAS-2B cell line derived from the human bronchial epithelium, referred in Ref. [31], using the forward primer GGAGCTCGTCTAGAGGATCCATGGGAGTCCCCAAGTTTTACAGATGG and the reverse primer GTCGAGGTCGACGGTATCCTCAGAAGGTTTAGAAACACCAAAATTAACAGCC and ligated into the pUG35 plasmid after the *MET17* promoter sequence using the unique restriction sites of BamHI and SalI. Both plasmids were transformed into the *xrn1*Δ strain (Y1544 and Y1470, respectively).

The pUG35-*MET17*(prom)-*GFP-URA3* plasmid was also used for exogenous expression of yeast *XRN1* C-terminus (encoding Xrn1_1133-1528_-GFP). **pUG35-*MET17*(prom)-*XRN1***_***1133-1528***_***-GFP-URA3*:** the C-terminal part (corresponding to amino acids 1133–1528 in the translated protein) of the native *XRN1* gene was amplified using the forward primer CTAGTGGATCCCCCGGGCATGAACATCGAAGGTCATGAAAGAAAAC and the reverse primer CGATAAGCTTGATATCGCGAGTAGATTCGTCTTTTTTATTATCACGGTCAGC and inserted into the pUG35 plasmid using the unique restriction sites of BamHI and HindIII downstream of the *MET17* promoter and upstream of the *GFP-*coding sequence, respectively. The plasmid was transformed into both wild-type and *xrn1*Δ strain (Y1487 and Y1488, respectively).

### 2.4 Confocal microscopy

Prior to imaging, 3 ml of liquid cell culture was centrifuged (1520 g for 3 min) and the supernatant was used as a solvent to prepare 1 % agarose that was left to solidify in a 35 mm diameter Petri dish. This was cut into four 10 × 10 mm blocks with a scalpel. Another 1 ml of the cell culture was concentrated by brief centrifugation (184 g for 1 min) and 1 μl of the concentrated cell suspension was loaded on standard No. 1.5 coverslip (Menzel) and immobilised by covering it with one of the 10 × 10 mm agarose blocks. The detailed step-by-step instructions can be found at protocols. io: https://www.protocols.io/view/live-cell-microscopy-sample-preparation-yeast-cult-8epv5r23dg1b/v1 and [32]. Microscopy images were acquired with a Zeiss LSM 880 confocal laser scanning microscope with an alpha Plan-Apochromat 100 × oil-immersion objective (NA = 1.46). The fluorescence signals of GFP (excited at 488 nm; argon laser) and mRFP (excited at 561 nm; solid-state laser) were detected using a photomultiplier tube detector with 493–550 nm and 540–660 nm emission filters, respectively. In all figures, individual transversal confocal sections are presented.

### 2.5 Glycolysis pathway and TORC1 signalling inhibition

To assess the effects of the initial steps of glycolysis on the subcellular distribution of Xrn1, cells from a 30-h culture were overlaid with a block of agarose (10 × 10 × 2 mm, i.e., 200 μl) as described above. Then, 3 μl of 10 % (w/v) D-glucose, 6-deoxy-D-glucose (6-DG) or 2-deoxy-D-glucose (2-DG) was added onto the top of the agarose block (final concentration of 0.15 %). Cells were imaged immediately after the addition of sugar and after 15 min. Alternatively, the 30-h culture was incubated with 1 mM iodoacetamide (IAA) for 30 min before sample preparation and the addition of glucose. To assess the requirement of TORC1 activation on the glucose-induced release of Xrn1 from the eisosome, a 28-h culture was incubated with 220 nM rapamycin for 2 h before sample preparation and glucose addition. All chemicals were purchased from Merck.

### 2.6 Microscopy image processing and data analysis

Image processing and analysis were performed in Fiji (ImageJ 1.54f) using custom-written macros used previously [[33], [34], [35]]. Subsequent processing of the analysis data, including the analysis of statistical significance of differences was performed using custom-written R scripts. The Fiji macros and R scripts (in the form of an R markdown) are available at: https://github.com/jakubzahumensky/microscopy_analysis. A detailed description of their use can be found in Ref. [32]. In brief, the cell segmentation masks used for the analysis were made using the Cellpose software [36] with parameters set in such a way that the mask edges intersected the plasma membrane patches in their middle. Incompletely imaged cells were removed automatically at this step. The segmentation masks were converted into regions of interest (ROIs) in Fiji, fitted with ellipses as approximations of cells, and curated manually to remove or adjust incorrectly created ROIs and remove ROIs of dead cells. For each cell, multiple parameters, including the mean and integrated fluorescence signal intensity (in the whole cell, plasma membrane, and cell interior) and cell cross-sectional area were automatically quantified. The mean values of parameters of interest were calculated from all cells within each biological replicate and respective condition. Statistical significance of differences was assessed by one-way ANOVA with Tukey’s honestly significant difference post-hoc test after testing for a normal distribution (Shapiro–Wilk test) and equal variance (Levene’s test). Graphing of the analysis results was performed using the Systat SigmaPlot 15 and Inkscape software.

## 3 Results

### 3.1 Eisosomal components Pil1 and Lsp1 mediate the Xrn1 binding to the eisosomes

**Fig. 1:**
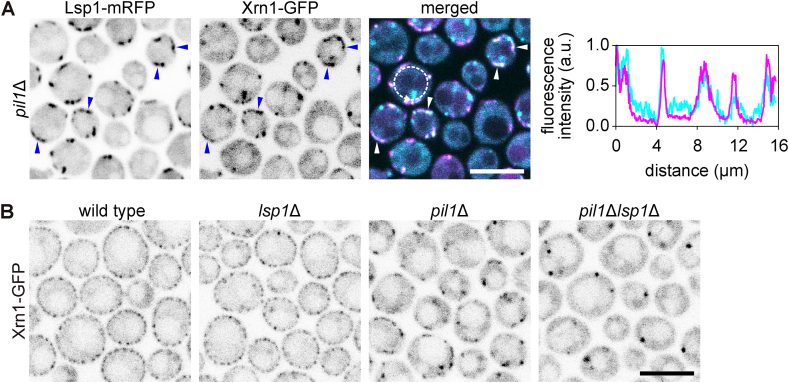
**Pil1 and Lsp1 mediate Xrn1 binding to eisosomes.** Localisation patterns of Lsp1-mRFP (magenta) and Xrn1-GFP (cyan) as detected in post-diauxic *pil1*Δ cells (strain Y1506) are shown in (**A**). Overlapping cortical accumulations (eisosome remnants) are highlighted with arrowheads. Profile of normalised fluorescence intensity (arbitrary units; right) along the plasma membrane of one cell (dashed arrow in merged image) indicates the degree of spatial correlation of both fluorescence signals. Subcellular localisation of Xrn1-GFP in post-diauxic cells of the wild-type (Y1288), *pil1*Δ (Y1286), *lsp1*Δ (Y1582) and *pil1*Δ*lsp1*Δ (Y1319) strains are compared in (**B**). Note the absence of cortical accumulations of Xrn1-GFP in *pil1*Δ*lsp1*Δ cells (right). Scale bars: 5 μm.

After the yeast cells of a growing culture have consumed all fermentable sugars from the medium, Xrn1 is sequestered at plasma membrane-associated eisosomes [11], leading to inhibition of its exoribonuclease activity [12]. In the absence of the eisosome organiser Pil1, eisosomes are reduced to so-called eisosome remnants, containing Lsp1 and other eisosomal proteins [25]. Under such circumstances, the sequestration of Xrn1 at the plasma membrane and the extent of its exoribonuclease activity inhibition are reduced [11,12]. When we analysed the subcellular distribution of GFP-labelled Xrn1 in more detail, we found that a significant fraction of the enzyme was associated with the plasma membrane even in *pil1*Δ cells. These cortical accumulations of Xrn1 colocalised with Lsp1-labelled eisosome remnants (Fig. 1A). This residual binding of Xrn1 to the plasma membrane was abolished when both eisosome organisers, i.e., Pil1 and Lsp1, were absent (Fig. 1B), and could be restored by exogenous expression of *PIL1* in *pil1*Δ*lsp1*Δ cells (Supplementary Fig. S1A). This indicates that the presence of at least one of the Pil1 or Lsp1 proteins is required for membrane sequestration of Xrn1. However, it is not sufficient to ensure this. Recently, we showed that a triple deletion mutant lacking the *PIL1* gene and the genes encoding two eisosome stabilisers, Seg1 and Nce102 (termed *snp*ΔΔΔ), is incapable of eisosome formation, analogous to the *pil1*Δ*lsp1*Δ strain [35]. Despite the presence of Lsp1, Xrn1 did not bind to the plasma membrane in the *snp*ΔΔΔ cells (Supplementary Fig. S1B). In agreement with previous studies that showed that the absence of Lsp1 alone does not substantially disrupt the regular distribution of eisosomes on the plasma membrane surface [25], we observed a wild type-like pattern of Xrn1 in the *lsp1*Δ strain (Fig. 1B).

### 3.2 SH3-like domain of Xrn1 is required for the eisosome-binding of Xrn1

**Fig. 2:**
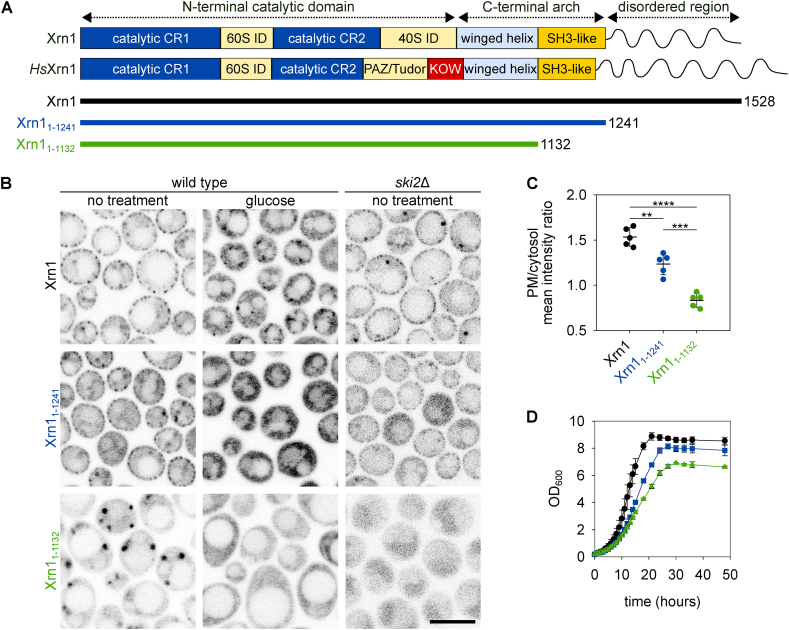
**SH3-like domain of Xrn1 is required for the eisosome-binding of Xrn1.** Domain architectures of the yeast Xrn1 and its human orthologue *Hs*Xrn1 are compared ((**A**); domain lengths are not to scale). The N-terminal catalytic domain is responsible for the exoribonuclease activity and is conserved among the Xrn family proteins. The C-terminal arch and the adjacent disordered C-terminal region represent regulatory domains. The coloured lines (bottom) represent full-length (black) and two truncated Xrn1 versions (Xrn1_1-1241_ – blue, and Xrn1_1-1132_ – green) prepared in this study. CR1/2 – conserved region 1/2, ID – interaction domain, PAZ – Piwi/Argonaute/Zwille; KOW – Kyprides, Ouzounis, Woese; SH3 – Src Homology 3. Subcellular localisation patterns of GFP-tagged full-length Xrn1 and its truncated analogues in post-diauxic untreated wild-type cells (left column in (**B**); strains Y1288, Y1439 and Y1440 in the upper, mid and lower row, respectively) and those treated with 2 % glucose (middle) are compared with those in (untreated) *ski2*Δ cells (right; strains Y1581, Y1432 and Y1433, respectively). Scale bar: 5 μm. The ratio of the mean fluorescence intensity at the plasma membrane (PM) to that in the cytosol was quantified in the indicated strains (**C**). The data are presented as the means ± SDs from 5 biological replicates (circles; 125–380 cells per condition). ∗∗, P ≤ 0.01, ∗∗∗, P ≤ 0.001, ∗∗∗∗, P ≤ 0.0001. One-way ANOVA with Tukey’s HSD (honestly significant difference) post-hoc test. Note the decrease in the PM/cytosol ratio to less than 1 in the strain expressing Xrn1_1-1132_, indicating the emergence of intensive protein accumulations in the cytosol. The growth curves of the full-length (black) and two C-terminally truncated Xrn1 versions (Xrn1_1-1241_ – blue, and Xrn1_1-1132_ – green) in *ski2*Δ-derived strains (Y1581, Y1432 and Y1433, respectively) are compared in (**D**). Data are represented as means ± SD from three independent biological replicates.

The *S. cerevisiae* Xrn1 is a large enzyme comprising 1528 amino acid residues with a molecular weight of 175.5 kDa. It is the major exoribonuclease in eukaryotes and is evolutionarily conserved from yeast to humans. The Xrn1 protein sequence consists of a conserved N-terminal catalytic domain, a basic middle domain termed C-terminal arch, specific for Xrn1 and its orthologues, and a disordered C-terminal region with a regulatory function [37,38]. The C-terminal arch contains a non-canonical *Src*-homology 3 (SH3) domain (hereafter referred to as the SH3-like domain; Fig. 2A). Canonical and non-canonical SH3 domains are involved in a range of protein-protein and protein-lipid interactions participating in cell signalling, proliferation, and other processes [39,40]. Based on this, we hypothesised that the SH3-like domain might be involved in the binding of Xrn1 to the eisosome. To test our hypothesis, we expressed two GFP-labelled C-terminally truncated Xrn1 mutants, one lacking only the disordered region (Xrn1_1-1241_) and the other lacking both the disordered region and the SH3-like domain (Xrn1_1-1132_; Fig. 2A). While Xrn1_1-1241_ partially retained the ability to bind to the eisosomes, Xrn1_1-1132_ was distributed exclusively in the cytosol, with occasional prominent focal accumulations (Fig. 2B). Analogous to the full-length Xrn1, Xrn1_1-1241_ was released from binding to the eisosome upon addition of glucose to the medium. Similarly, internal bodies enriched with the Xrn1_1-1132_ protein disappeared in the presence of glucose and Xrn1_1-1132_ homogenously distributed in the cytosol (Fig. 2B). The membrane binding of the Xrn1 variants was quantified [32] in terms of the ratio of the mean fluorescence intensity at the plasma membrane (PM) to that in the cytosol. The membrane fraction of Xrn1 decreased in both mutant strains, to a greater extent in the strain expressing Xrn1_1-1132_ (Fig. 2C). The cells of this strain were enlarged compared to those of the wild-type, which could indicate a defect in exoribonuclease function [37]. To test whether the truncated Xrn1 mutants were catalytically active, we conducted a synthetic lethality test. Specifically, we expressed the mutant Xrn1 variants in a strain lacking the *SKI2* gene. As Ski2 is required for 3’-5’ mRNA decay, effective 5’-3’ exoribonuclease activity is essential for *ski2*Δ cell survival. The expression of the truncated Xrn1_1-1241_ or Xrn1_1-1132_ instead of full-length Xrn1 was sufficient to ensure the growth of *ski2*Δ cells, indicating that both truncations were at least partially catalytically active (Fig. 2B–D).

SH3 domains preferentially bind to proline-rich motifs (PRMs) [40]. Notably, the N-terminal sequences of the major eisosomal components Pil1 and Lsp1 contain a PRM (five prolines P21-P25) that is not involved in eisosome formation [24]. This makes the PRMs of Pil1 and Lsp1 suitable candidates for physical interaction with other proteins. To test whether this region is necessary for the eisosomal binding of Xrn1, we expressed a mutant Pil1 protein, in which the PRM (P21-P25) was replaced with the flexible linker sequence GSGSG (Pil1(5P)), in the *pil1*Δ*lsp1*Δ background. In the *pil1*Δ*lsp1*Δ cells, all the eisosomes were assembled exclusively from Pil1(5P). If the PRM is essential for eisosomal Xrn1 binding, Xrn1 segregation should be disrupted. However, this was not the case, as normal eisosomal segregation of Xrn1 occurred at the Pil1(5P) eisosomes (Supplementary Fig. S2). We concluded that if they are involved at all, the PRMs in the eisosome organisers Pil1 and Lsp1 are not necessary for Xrn1 binding.

Finally, we asked whether the SH3-like domain is sufficient for the Xrn1 binding to the eisosome. To answer this, we fused the C-terminal part of the Xrn1 molecule including the SH3-like domain with the GFP tag and localised this fusion protein in post-diauxic cells. Independent of the presence of the chromosomally-encoded unlabelled full-length Xrn1, Xrn1_1133-1528_-GFP did not localise to the eisosome. While it remained completely cytosolic in *xrn1*Δ cells, it concentrated at internal bodies when expressed in the wild-type background (Supplementary Fig. S3). Our results showed that the SH3-like domain is not sufficient to target Xrn1 to the eisosome.

### 3.3 Glycolysis inhibition induces eisosomal binding of Xrn1

**Fig. 3:**
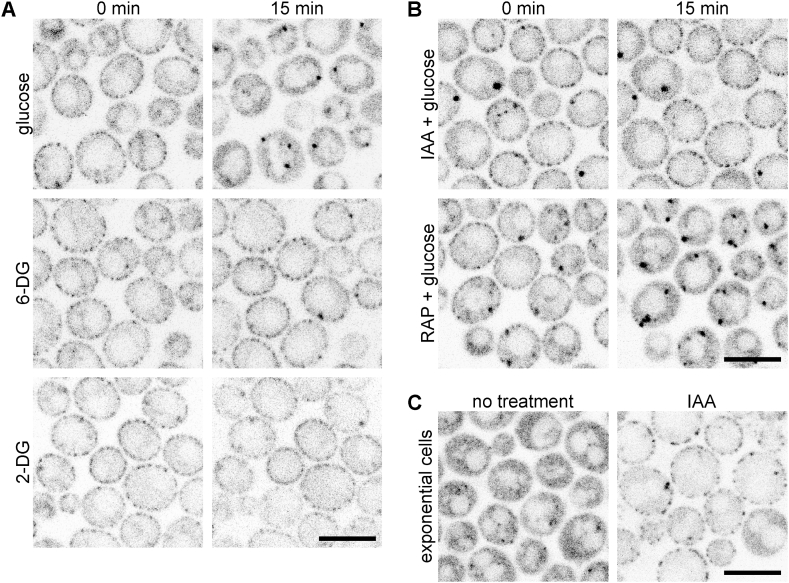
**Glycolysis inhibition induces eisosomal binding of Xrn1.** Subcellular distributions of Xrn1-GFP in the wild-type (Y1288) post-diauxic cells at times 0 and 15 min after the addition of D-glucose, 6-deoxy-D-glucose (6-DG) or 2-deoxy-D-glucose (2-DG) are shown in (**A**), see Methods for details. In the same arrangement, post-diauxic wild-type (Y1288) cells expressing *XRN1-GFP*, pre-treated either with 1 mM iodoacetamide (IAA) for 30 min or 220 nM rapamycin (RAP) for 2 h were supplied with glucose (**B**). The same field of view was imaged at both indicated times in A and B. Late-exponential wild type cells expressing Xrn1-GFP (strain Y1288) before and after the treatment with 1 mM IAA for 2 h are shown in (**C**). Scale bars: 5 μm.

Upon the addition of glucose to a population of post-diauxic cells, Xrn1 is rapidly released from eisosomes and is homogeneously distributed in the cytosol [11]. To better understand the actual signal triggering the Xrn1 release, we targeted various steps of glycolysis. First, we probed the involvement of the initial glycolysis steps, i.e., glucose transport into the cell and the phosphorylation at the C6 position. We supplied the cells with two glucose analogues, 6-deoxy-D-glucose (6-DG) and 2-deoxy-D-glucose (2-DG), which are readily imported into the cells. However, 6-DG cannot be phosphorylated by the hexokinase and thus cannot pass through the first step of glycolysis. 2-DG can be phosphorylated at C6, forming 2-deoxy-D-glucose-6-phosphate, but cannot be subsequently phosphorylated at C1, resulting in the halting of glycolysis ([41] and references therein). Neither of these glucose analogues caused Xrn1 release from the eisosomes of post-diauxic cells (Fig. 3A), indicating that rather than glucose sensing, import and phosphorylation at C6, further glycolysis steps are required to trigger the Xrn1 release from the eisosome.

We then analysed the effect of pre-treating the cells with iodoacetamide (IAA), which is frequently used to inhibit glycolysis. Specifically, IAA has been proposed to irreversibly inhibit glyceraldehyde-3-phosphate dehydrogenase (GAPDH) at the sixth step of glycolysis [42]. Supplying the IAA-treated cells with glucose did not evoke the release of Xrn1 from the eisosome (Fig. 3B). In addition, the eisosomal sequestration of Xrn1 was induced when a late-exponential yeast culture was treated with IAA (Fig. 3C). Our results indicated that the signal for Xrn1 release from the eisosome is due to ongoing glycolysis, presumably following the GAPDH action.

As active glycolysis stimulates TORC1 signalling [41,43], we tested whether activation of TORC1 is involved in the release of Xrn1 from the eisosome upon glucose addition. However, pre-treatment of the post-diauxic culture with rapamycin, a well-known TORC1 inhibitor [43], did not prevent the Xrn1 release (Fig. 3B), indicating that TORC1 activation is dispensable in this process.

### 3.4 Nutrition-dependent aggregation of Xrn1 is conserved from yeast to humans

Xrn1 is evolutionarily conserved in eukaryotes ranging from unicellular yeast to mammals (Fig. 2A) [44]. Xrn1 conditionally aggregates in the neurons of a rodent model in the form of synaptic Xrn1 (SX) bodies [45]. The SX bodies form following the stimulation of NMDA (N-methyl-D-aspartate) receptors and are subsequently dissolved upon the activation of metabotropic glutamate receptors. The formation of SX bodies, which are deemed to have an mRNA-protective and/or storing function, is inversely correlated with a reduction in the local translation rate [45]. Similarly, in yeast, Xrn1 accumulates at the eisosome under conditions of decreased cell proliferation, which correlates with lower overall protein synthesis. At the same time, the Xrn1-mediated degradation of the translation template, mRNA, is inhibited upon the enzyme segregation at the eisosomes. A comparison of the Xrn1 aggregates formed in yeast and mammals suggests that there may be a conservation of Xrn1 aggregate formation, which may serve as a novel mechanism of Xrn1 activity modulation, involved in the regulation of overall gene expression [45,46].

**Fig. 4:**
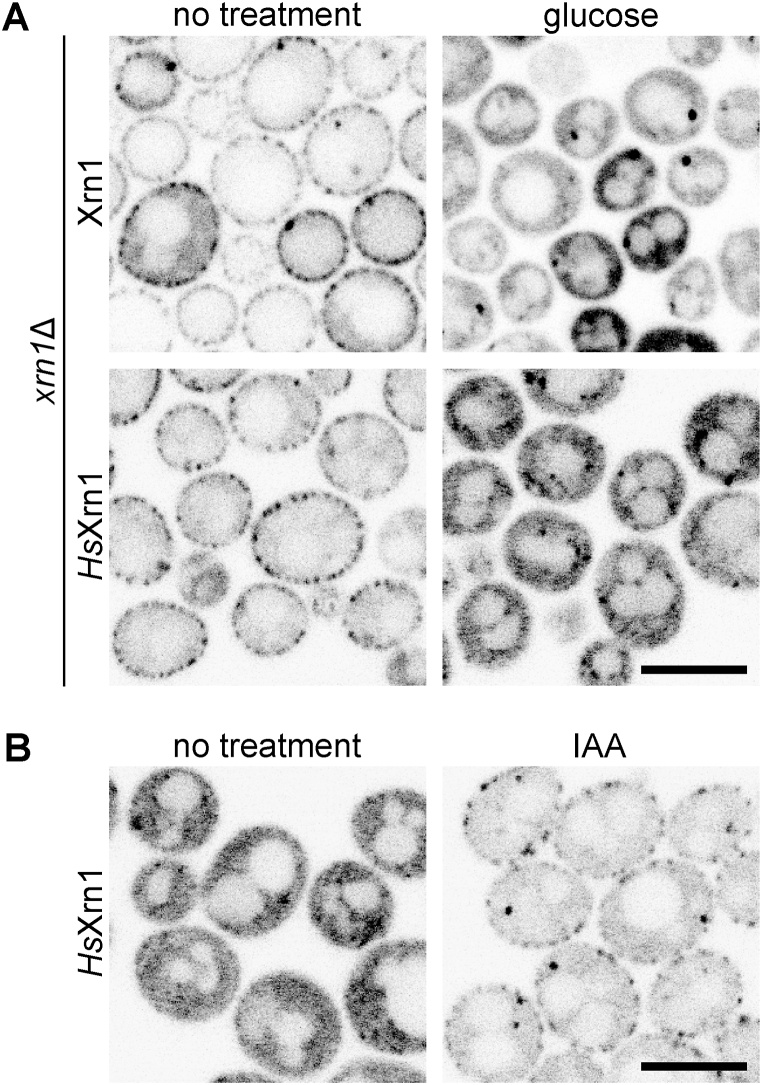
***Hs*Xrn1 localises like its yeast homologue under conditions of halted glycolysis when expressed in yeast.** Distribution patterns of exogenously expressed GFP-tagged native yeast Xrn1 and human Xrn1 (*Hs*Xrn1-GFP) in the *xrn1*Δ background (strains Y1544 and Y1470, respectively) in post-diauxic cells before and after the supply of 2 % glucose to the medium are compared in (**A**). Localisation of exogenously expressed *Hs*Xrn1-GFP in late-exponential *xrn1*Δ (Y1470) cells treated with nothing or 1 mM iodoacetamide (IAA) for 1 h is shown in (**B**). Scale bars: 5 μm.

To test this hypothesis, we expressed GFP-tagged human Xrn1 (*Hs*Xrn1) in *xrn1*Δ yeast from a centromeric plasmid. Like the yeast Xrn1, heterologously expressed *Hs*Xrn1 underwent glycolysis-dependent changes in subcellular distribution and was sequestered at the eisosomes in the post-diauxic phase (Fig. 4A). Analogous to its yeast counterpart, the eisosomal segregation of *Hs*Xrn1 could be induced even in the presence of glucose when glycolysis was arrested by IAA treatment (Fig. 4B). Our findings are consistent with the hypothesis that the reversible segregation of Xrn1 is an evolutionarily conserved feature in eukaryotes and serves as a nutrition-dependent regulatory mechanism of gene expression.

## 4 Discussion

In this report, we addressed the molecular principles of eisosomal binding and activity regulation of the conserved exoribonuclease Xrn1. In agreement with previous observations [11,12], we found decreased plasma membrane binding of the protein in *pil1*Δ cells. Using the fluorescently labelled Pil1 homologue Lsp1, we identified the small number of bright accumulations of the Xrn1-GFP signal in the cortex of *pil1*Δ cells with eisosome remnants (Fig. 1A). In the complete absence of eisosomes, Xrn1 became exclusively cytosolic, independent of whether Lsp1 was present (in the *snp*ΔΔΔ strain, Supplementary Fig. S1B) or not (*pil1*Δ*lsp1*Δ, Fig. 1B). In *lsp1*Δ cells, Xrn1 was distributed similarly to the wild type. This is consistent with the negligible effect of Lsp1 absence on the eisosome morphology reported previously [25]. Consistent with recently reported biochemical association between Xrn1 and eisosomal components [47], these data imply that a cortical protein scaffold provided by the eisosome [24], or its remnants, is required for the plasma membrane binding of Xrn1.

Our results show that the SH3-like domain localised in the C-terminal arch of the Xrn1 protein is likely involved in this binding, since its removal prevented the cortical localisation of the protein (Fig. 2). However, a PRM in the N-terminal region of Pil1, which we suggested to be a potential binding partner for the SH3-like domain of Xrn1, was dispensable for Xrn1 accumulation at the eisosome (Supplementary Fig. S2). Another PRM is present in the C-terminal disordered region of the Xrn1 molecule itself (five prolines P1445-P1449). It has been speculated previously that it may play a role in Xrn1 aggregation [45]. However, the truncated version Xrn1_1-1241_, which contained the SH3-like domain but not the PRM, retained its eisosomal localisation, albeit to a reduced extent (Fig. 2B). In general, the affinity of tested Xrn1 versions to the eisosome was lower in *ski2*Δ cells (Fig. 2B). This is consistent with a previous report that showed that Xrn1 is inactive when bound to the eisosome [30]. Viability of *ski2*Δ cells critically depends on 5’-3’ mRNA degradation executed by Xrn1. Therefore, complete eisosomal sequestration of the enzyme would be lethal in this case.

Although required for eisosomal binding of Xrn1, the SH3-like domain was not sufficient to target Xrn1_1133-1528_-GFP there. However, a clear difference between the localisation patterns of Xrn1_1133-1528_ in the presence or absence of the native full-length Xrn1 suggested another important thing. Accumulation of Xrn1_1133-1528_ in cytosolic bodies when unlabelled full-length Xrn1 expressed from a chromosomal locus is present (Supplementary Fig. S3) could indicate its binding to a sequence of the Xrn1 molecule upstream of the SH3-like domain. That would not be surprising, as it has previously been suggested that parts of the sequence of the Xrn1 molecule located upstream and downstream of the SH3-like domain interact with each other to regulate the exoribonuclease activity of the protein [48,49]. However, it also indicates that if this binding plays any role in the subcellular distribution of the protein, it alone is not sufficient to move the protein from the P-body to the eisosome.

Physical interaction of Xrn1 with the eisosomal core components Pil1 and Lsp1 has been suggested in a large-scale study [50] and confirmed in a targeted search for protein interaction partners of Xrn1 in post-diauxic yeast culture [47]. However, further data will be needed to elucidate the molecular principle of the binding. Specifically, Pil1 and Lsp1 may directly present specific amino acid sequences/motifs that can be bound by Xrn1. Alternatively, the eisosome scaffold may establish a suitable environment for either presenting a binding site or attracting the actual interactor for the SH3-like domain of Xrn1. The proof of the first possibility, in particular, is challenging, as the eisosome is rather sensitive to not only targeted mutations of three specific regions of Pil1 and Lsp1 directly involved in scaffold cross-linking or other regions responsible for binding specific lipids, phosphatidylinositol-4,5-bisphosphate, ergosterol, and phosphatidylserine [24], but also to interference with several phosphorylation sites in Pil1 and Lsp1 molecules [51,52]. Changes in any of these parameters can lead to eisosome destabilisation, making it difficult to assess the causes of reduced Xrn1 association with the plasma membrane under these conditions.

Previous studies concluded that Xrn1 is released into the cytosol upon glucose addition [11,12]. We show that the delivery of a fermentable carbon source into the cell is by itself not sufficient for this release. Each of the glucose analogues tested (6-DG and 2-DG) is successfully recognised and transported into the cytosol but is unable to trigger Xrn1 release (Fig. 3), even though the latter undergoes the initial glycolytic reaction. Thus, the Xrn1 release appears to be triggered by further fermentation steps, as non-fermentable carbon sources like glycerol do not elicit it either [11]. In support of the fermentation requirement, we found that the inhibition of glycolysis by iodoacetamide (IAA) not only prevented the glucose-induced release of Xrn1 from the eisosome but also triggered its binding to the eisosome in the late-exponential phase, i.e., prior to glucose depletion (Fig. 3).

Although IAA is thought to specifically inhibit the sixth step of glycolysis, it does so by irreversibly alkylating catalytic cysteine residues of glyceraldehyde-3-phosphate dehydrogenase, which indicates possibly broader, rather non-specific mode of action [42]. Thus, although Xrn1 binding to the eisosome is linked to glucose metabolism, the involvement of other metabolic and/or signalling pathways cannot be ruled out. Among the first to be explored is the role of downstream effectors of glycolysis. One of the signalling pathways tightly bound to glycolysis is TORC1, the master regulator of cell proliferation. Interestingly, the rate of TORC1 activity negatively correlates with Xrn1 binding to the eisosome – Xrn1 is localised diffusely in the cytosol in exponential cells where TORC1 is highly active and bound to the eisosome in the post-diauxic phase when TORC1 exhibits only a basal activity [11,41,43]. A recent study reported that there are at least three distinct mechanisms of glucose metabolism-induced activation of TORC1, the first of which is mediated by glucose-6-phosphate [41]. However, the supply of 2-DG, although successfully phosphorylated at C6 and thus activating TORC1 [41], was not sufficient to trigger the release of Xrn1 from the eisosome (Fig. 3A). In addition, inhibiting TORC1 with rapamycin did not prevent the glucose-triggered release of Xrn1 from the eisosome in post-diauxic cells (Fig. 3B). These two observations indicate that the sequestration of Xrn1 at the eisosome is not regulated by TORC1 signalling.

Regardless of the mechanism by which Xrn1 binds to the eisosome, our results have yielded another important finding: this mechanism works identically for yeast and human Xrn1 proteins. We report that when expressed in yeast, the human *Hs*Xrn1 binds to the eisosome upon depletion of fermentable carbon sources, analogous to the native yeast protein (Fig. 4). Although we did not test the effect of this binding on the activity of the enzyme, we can reasonably assume that the activity decreases, since the enzyme is effectively removed from the rest of the mRNA decay machinery and the mRNAs themselves [11].

It has been reported that Xrn1 in rat hippocampal neurons aggregates into synaptic Xrn1 (SX) bodies following the activation of NMDA receptors. The question of plasma membrane binding of SX bodies has not been addressed [45], but we cannot exclude this option and actually deem it likely. Similar to the eisosome binding in yeast, other proteins of the mRNA decay machinery do not localise to these protein aggregates. This means that Xrn1 is likely inactive when in SX bodies, even though available data indicate that SX bodies, unlike eisosomes, accumulate mRNAs, which can be translated once released [45]. Therefore, while there are some similarities between Xrn1 binding to SX bodies and eisosomes, the functional implications seem to diverge. Nevertheless, the fact that the formation of aggregates of yeast and human Xrn1 protein is driven by the same metabolic cues strikes us as an important finding that merits further investigation in the future.

## Data availability

The raw and processed image data, and the data of the image analysis (regions of interest, results of quantification and statistical analyses; Fig. 2C) have been deposited in Zenodo: https://zenodo.org/records/12748898. The code used for microscopy image analysis is part of a preprint by Zahumensky & Malinsky [32] and has been deposited on GitHub: https://github.com/jakubzahumensky/microscopy_analysis.

## Funding

This study was supported by the 10.13039/501100001824Czech Science Foundation, grant number 20-04987S; and institutional support. Microscopy was performed at the Microscopy Service Centre of the Institute of Experimental Medicine CAS supported by the 10.13039/501100001823MEYS CR (LM2023050 Czech-Bioimaging). S. Mondal was financially supported by the scholarship provided by the Faculty of Science of Charles University. P. Vesela was financially supported by the Czech Academy of Sciences Postdoctoral fellowship (L200392451).

## CRediT authorship contribution statement

**Satyendra Mondal:** Writing – review & editing, Writing – original draft, Visualization, Investigation, Formal analysis, Data curation, Conceptualization. **Jakub Zahumensky:** Writing – review & editing, Writing – original draft, Visualization, Validation, Software, Methodology, Formal analysis, Data curation. **Petra Vesela:** Writing – review & editing, Validation, Resources. **Jan Malinsky:** Writing – review & editing, Writing – original draft, Supervision, Resources, Project administration, Methodology, Funding acquisition, Conceptualization.

## Declaration of competing interest

The authors declare that they have no known competing financial interests or personal relationships that could have appeared to influence the work reported in this paper.
