# Supplementary material for: Conserved mechanism of Xrn1 regulation by glycolytic flux and protein aggregation
Source: Heliyon. 2024 Oct 1;10(19):e38786. doi: 10.1016/j.heliyon.2024.e38786 (PMC11481674; doi:10.1016/j.heliyon.2024.e38786)
Supplement: Multimedia component 1 [file mmc1.docx]

**Supplementary Information**


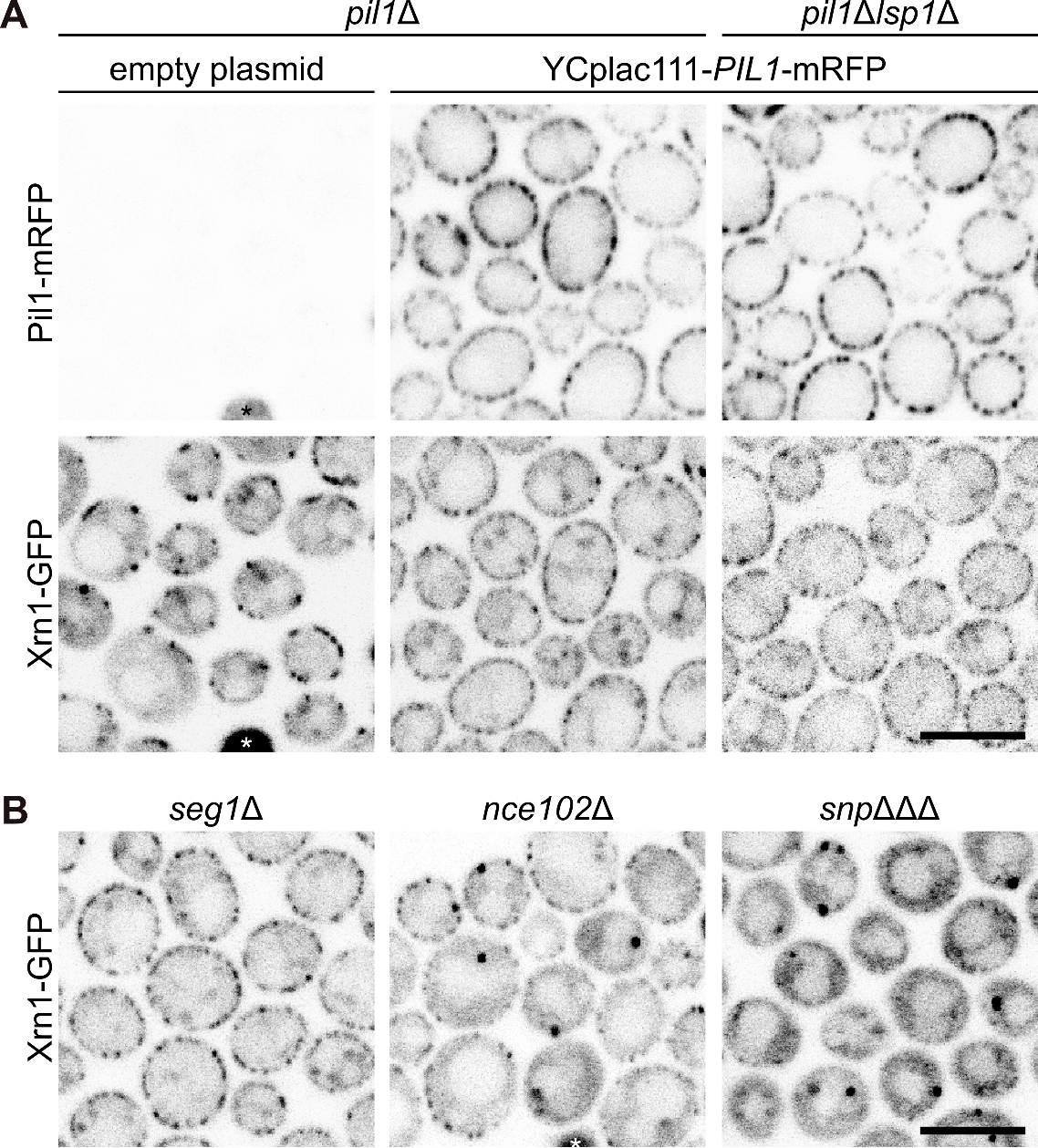


**Supplementary Figure S1. Xrn1 binding to the plasma membrane requires an eisosome(-like) protein scaffold.** Subcellular distributions of Xrn1-GFP and Pil1-mRFP in post-diauxic *pil1*∆ (Y1451) and *pil1*∆*lsp1*∆ (Y1565) cells exogenously expressing Pil1-mRFP from a centromeric plasmid under its native promoter are compared in (**A**). A control localisation experiment was performed under the same conditions in the *pil1*∆ (Y1449) strain expressing the empty plasmid YCplac111-*mRFP*. Localisation patterns of Xrn1-GFP in post-diauxic cells of the *seg1*∆ (Y1490), *nce102*∆ (Y1452), and *seg1*∆*nce102*∆*pil1*∆ (*snp*∆∆∆; Y1453) background strains are presented in (**B**). Asterisks denote dead cells. Scale bars: 5 µm.


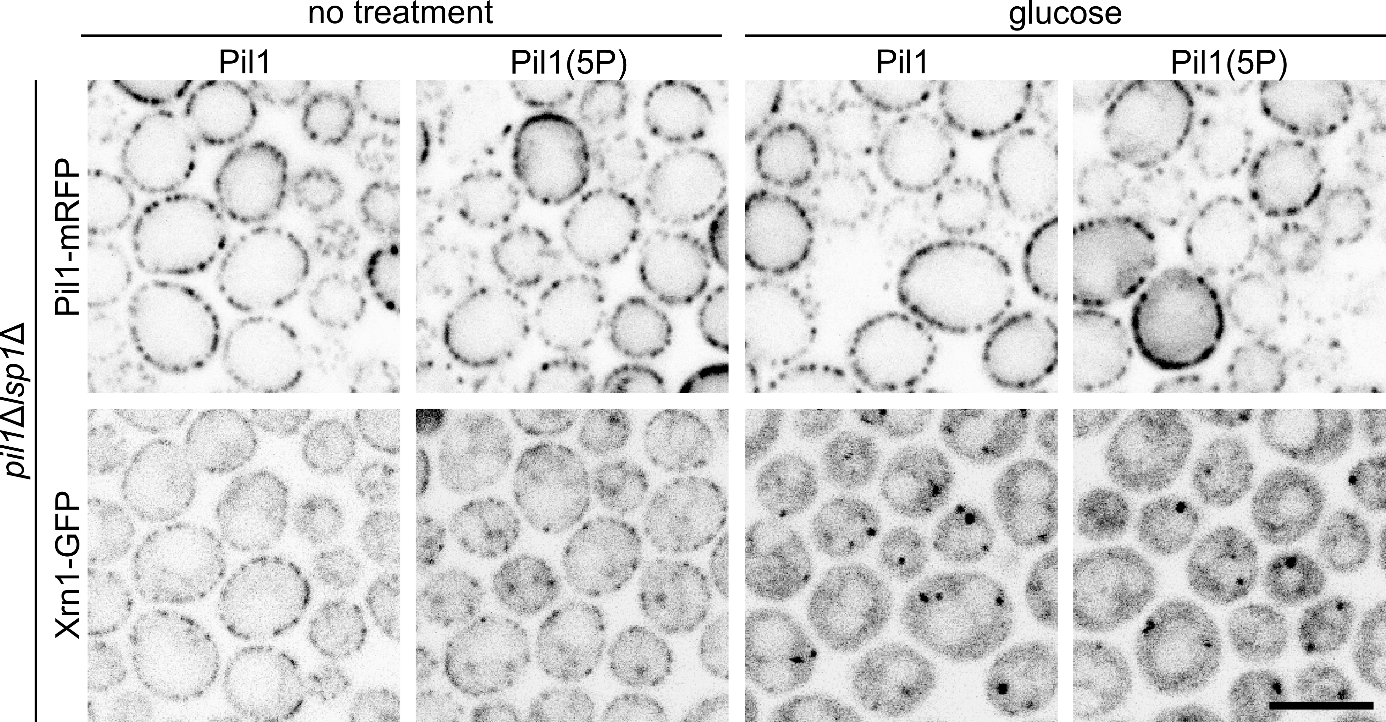


**Supplementary Figure S2. Xrn1 accumulates at eisosomes formed by Pil1(5P).** Subcellular distributions of Xrn1-GFP in post-diauxic *pil1*∆*lsp1*∆ cells expressing either Pil1-mRFP (strain Y1565) or a PRM mutant Pil1(5P)-mRFP (Y1578) from the centromeric plasmid YCplac111 were monitored before (no treatment) or after 2% glucose was added to the medium (glucose). Scale bar: 5 µm.


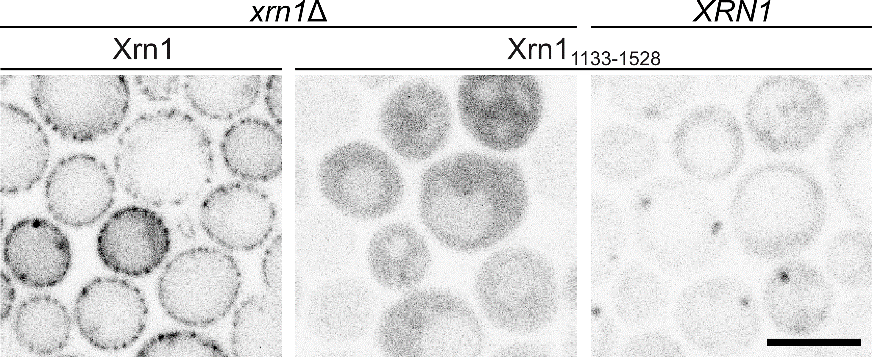


**Supplementary Figure S3:** **Localisation of GFP-tagged C-terminal part of Xrn1.** Localisation patterns of Xrn1_1133-1528_-GFP in the wild-type (Y1487) and *xrn1*∆ mutant (Y1488) were compared with that of the full-length protein (Y1544). Cells were cultivated in synthetic Ura- dropout medium for 30 hours as described in Methods. Scale bar: 5 µm.
